# Clinical characteristics, management, and outcomes of patients with primary cardiac angiosarcoma: A systematic review

**DOI:** 10.34172/jcvtr.2023.30531

**Published:** 2023-03-16

**Authors:** Diego Chambergo-Michilot, Gabriel De la Cruz-Ku, Rosalie M. Sterner, Ana Brañez-Condorena, Pedro Guerra-Canchari, John Stulak

**Affiliations:** ^1^Universidad Científica del Sur, Lima, Perú; ^2^Department of Cardiology Research, Torres de Salud National Research Center, Lima, Perú; ^3^Department of Surgery, Mayo Clinic, Rochester, MN, USA; ^4^Department of Surgery of the University of Massachusetts Medical School, Worcester, MA, USA; ^5^Universidad Nacional Mayor de San Marcos, Facultad de Medicina, Lima, Perú; ^6^Asociación de Investigación Estudiantil en Ciencias de la Salud, Lima, Perú; ^7^Sociedad Científica de San Fernando, Lima, Perú

**Keywords:** Hemangiosarcoma, Angiosarcoma, Malignant Haemangioendothelioma, Heart, Systematic review

## Abstract

Primary cardiac angiosarcomas (PCA) are highly aggressive malignant heart tumors. Previous reports have shown a poor prognosis regardless of management, and no consensus or guidelines exist. It is necessary to clarify this information since patients with PCA have a short survival. Therefore, we aimed to systematically review clinical manifestations, management, and outcomes. We systematically searched in PubMed, Scopus, Web of Science, and EMBASE. We intended to include cross-sectional studies, case-control studies, cohort studies, and case series that reported clinical characteristics, management, and outcomes of patients with PCA. As a methodological approach, we used the Joanna Briggs Institute Critical Appraisal Checklist for Case Series and the Newcastle-Ottawa Scale for cohorts. We included six studies (five case series, one cohort). The mean/median age ranged from 39 to 48.9 years. Male sex was predominant. The most frequent manifestations were dyspnea (range: 50%-80%), pericardial effusion (29% & 56%), and chest pain (10%-39%). The mean tumor size ranged from 5.8 to 7.2 cm, with the majority of these localized in the right atrium (70-100%). The most common locations of metastasis were the lung (20%-55.6%), liver (10%-22.2%), and bone (10%-20%). Resection (22.9%-94%), and chemotherapy as neoadjuvant or adjuvant (30%-100%) were the most commonly used methods of treatment. Mortality ranged from 64.7% to 100%. PCA often presents late in its course and usually results in poor prognosis. We strongly recommend performing multi-institutional prospective cohorts to better study disease course and treatments to develop consensus, algorithms, and guidelines for this type of sarcoma.

## Introduction

 Cardiac malignant tumors are a group of infrequent neoplasms with high mortality.^1-3^ Moreover, the majority (94.3%) are primary cardiac tumors (PCT). Malignant PCTs (MPCTs) have higher mortality than benign PCTs, ^2^ and they commonly affect women at an early age of diagnosis.^4,5^

 Most frequent MPCT are sarcomas (62-65%).^5,6^ A prior study reported a survival of 46%, 22%, and 17% at one, three, and five years of follow-up, respectively, in this group.^5^ Risk factors for early mortality are old age ( ≥ 80 years), non-surgical treatment, and certain histopathological types, such as angiosarcomas.^5,7^ Angiosarcomas are the most frequent specific MPCT, accounting for more than 50% of sarcomas.^5^ In terms of primary cardiac angiosarcomas (PCA), one study reported that sex was equally distributed, the mean age of diagnosis was 44.4 years, and the right atrium was the most common location.^8^

 About diagnosis, it has been demonstrated the utility of magnetic resonance imaging, which describes a cauliflower-like lesion.^9^ With regards to the management of PCA, a previous 20-year experience showed that most patients with PCA underwent surgery and chemotherapy, and these approaches were associated with an increase in survival.^10^ However other studies did not report any benefit, maybe due to the low number of patients.^11,12^ There are no consensus or guidelines about PCA management. It is necessary to clarify this information since patients with PCA have a poor prognosis and overall mean survival of less than one year.^9-11^

 Therefore, we aimed to perform a systematic review of observational studies to synthesize evidence on PCA clinical manifestations, management, and prognosis to make evidence-based decisions that improve health care in patients with PCA.

## Material and Methods

 This systematic review followed the recommendations of the Preferred Reporting Items for Systematic Reviews and Meta-analyses (PRISMA) statement.^13^ The protocol was registered in PROSPERO (CRD42020191063) and Figshare.^14^

###  Search strategy 

 We searched evidence up to August 10^th^, 2020 in the following databases: PubMed, Scopus, Web of Science, and EMBASE. The search strategies are available in the Supplementary Material 1. We did not limit the search by publication date or language.

###  Inclusion criteria

 We intended to include cross-sectional studies, case-control studies, cohorts, and case series that reported clinical characteristics, management, and outcomes of patients with PCA. We excluded trials, reviews, letters to the editor, congress or conference abstracts, case reports, editorials, interviews, comments, and newspaper articles.

###  Study selection

 One author (DCM) downloaded all found references to an EndNote library and eliminated duplicates. Then, the author exported those references to the Rayyan webpage (https://rayyan.qcri.org/). Two authors (DCM and PGC) independently screened titles and abstracts to select potential studies for inclusion. Finally, those authors assessed the full-text version of each potential study to determine eligibility. Any disagreement was discussed and resolved by consensus.

###  Data extraction

 Two authors (DCM and PGC) independently extracted data of interest. For dichotomous outcomes, we extracted relative frequencies. The extraction was performed using a pre-piloted Microsoft Excel sheet. Any disagreement was discussed and resolved by consensus. When there were doubts about any missing information in the studies, we sent emails to the authors to clarify the information.

###  Methodological assessment

 Two authors (ABC and PGC) assessed the quality of eligible studies independently. Five out of the six included studies were case series; hence we used the Joanna Briggs Institute Critical Appraisal Checklist for Case Series.^15^ This tool consists of 10 questions about inclusion criteria, condition identification, reporting of demographic and clinical information, and statistical analysis. Possible answers are “Yes”, “No”, “Unclear” or “Not/Applicable”. Previous systematic reviews have used this tool to assess case series.^16^

 We used the Newcastle-Ottawa Scale (NOS) for one study, which was a cohort. This tool assesses three domains: selection, comparability of groups, and ascertainment of the outcome. A score ≥ 7 meant low risk of bias, a score of 4-6 meant high risk of bias, and a score < 4 meant very high risk of bias.^17^ Any disagreement was discussed and resolved by consensus.

###  Data synthesis

 Characteristics of patients were reported as frequencies, mean (standard deviation, SD) or median (interquartile range) according to how the authors reported their results. We performed a descriptive approach of the frequency of clinical manifestations. We synthesized clinical manifestations, management, and outcomes of patients with PCA in each study.

## Results

**Figure 1:**
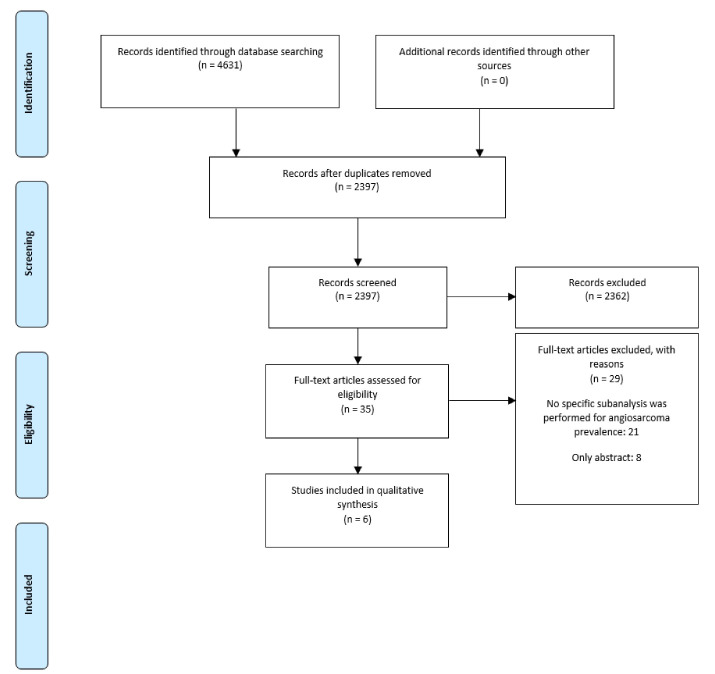
PRISMA Flowchart of selection

 After duplicate removal, we screened 2,397 records. Finally, we included six studies in the qualitative synthesis (Figure 1). One manuscript was a cohort study, and the rest were case series. The sample size ranged from 9 to 168 patients. The mean/median age ranged from 39 to 48.9 years. In four studies, more than a half were male (55.6%-78%) (Table 1).

**Table 1:** Clinical characteristics of population of included studies

**Study**	**Design**	**Sample size**	**Age**	**Male**	**Main manifestations**	**Ventricular function**	**Hemodynamics**	**Size (cm)**	**Location**	**Extension**	**Histological grade **	**Immunohistochemical markers**
Yu, 2019 ^11^	Case series	9	48.9 ^1^	55.6%	Chest pain, vomiting, cough, hemoptysis, dyspnea, pericardial/pleural effusions, and fatigue	NR	NR	5.8 ^1^	RA: 100%	RV: 22.2%	NR	CD31 + , CD34 + , Fli-1 + , CD117 + , SMA-, Desmin-, S-100-, CKpan-, Ki-67 +
Zhang, 2019 ^8^	Cohort	168	44.4 ± 15.5 ^1^	56%	NR	NR	NR	> 5 cm: 37.5%	NR	Extension to nodes Localized: 19.6% Regional: 26.2% Distant: 51.8%	I: 0.6% II: 3% III: 15.5% IV: 17.3% ^4^	NR
Leduc, 2017 ^19^	Case series	10	47.8 ± 17.1 ^1^	40%	NR	NR	NR	6.4 ± 2.8 ^1^	RA: 70%	NR	NR	CD31 + , CD34 +
Kupsky, 2016 ^12^	Case series	17	46 (range: 23-77) ^2^	35.3%	Dyspnea (59%), recurrent pericarditis/pericardial effusion (29%), pleuritic chest pain (24%), cardiac tamponade (18%), and chest pressure (12%)	Normal LVEF: 94% Normal RVEF: 88.2%	Flow obstruction: 23.5%	6.45 (4.4-8.1) ^2,3^	RA: 76.47%	Pericardium: 71%	NR	NR
Look Hong, 2012 ^10^	Case series	18	39 (range: 24-70) ^2^	78%	Pericardial effusion (56%), dyspnea (50%), chest pain (39%), syncope (28%), hemoptysis (17%), cardiac arrest (5%)	NR	NR	5.9 ^1^	RA: 89%	Pericardium: 17% Atrioventricular groove: 17%	I: 5% II: 22% III: 39%	CD31 + , CD34 +
Ge, 2011 ^18^	Case series	10	40 (range: 20- 61)^1^	60%	Dyspnea (80%), fatigue (20%), cough (20%), chest pain (10%)	NR	NR	7.2 ^1^	RA: 100%	RCA: 70% RV: 60%	II: 40% III: 60%	CD31 + , CD34 + , Fli-1 + , WT-1 + , Ki 57, p53

Abbreviations: RA, right atrium; RV, right ventricle; RCA, right coronary artery; LVEF, left ventricular ejection fraction; RVEF, right ventricular ejection fraction; NR, not reported ^1^ Mean ± standard deviation ^2^ Median (range) ^3^ Calculated using Stata v14. ^4^ The rest were unknown

 The most frequent clinical manifestations included dyspnea (3 studies: 50%, 64.7%, & 80%), pericardial effusion (2 studies: 29% & 56%) and chest pain (3 studies: 10%, 23.5%, & 39%).^10,12,18^ One study reported that left and right ventricular function were normal; however, flow obstruction accounted for the 23.5%.^12^ The mean tumor size ranged from 5.8 to 7.2 cm, and the right atrium was the most frequent location (70-100%). Extension location was heterogeneous among studies: pericardium (2 studies: 17% & 71%)^10,12^ and right ventricle (2 studies: 22.2% & 60%)^11,18^ were relatively frequent. One study reported that extension to regional and distant lymph nodes accounted for 78%.^8^ Three studies reported that high histological grade was frequent among PCA (15.5%-60%).^8,10,18^ Regarding biomarkers, the most commonly used were CD31 + , CD34 + and Fli-1 + (Table 1).

 Resection was a frequent treatment option (22.9%-94%); however, its use is dependent on clinical status and staging. For instance, Yu et al^11^ declared that only patients with small tumors received resection, and the rest did not due to extensive metastases. Chemotherapy, as neoadjuvant or adjuvant, was frequently utilized as well (30%-100%). One study reported the regimens, which were MAID (mesna, doxorubicin, ifosfamide, dacarbazine), gemcitabine/docetaxel, liposomal doxorubicin, liposomal doxorubicin/paclitaxel, doxorubicin/ifosfamide/mesna, and doxorubicin/dacarbazine.^10^ (Table 2).

**Table 2:** Outcomes and treatment of population of included studies

**Study**	**Resection**	**Chemotherapy**	**Radiotherapy**	**Chemoradiotherapy**	**Metastasis**	**Follow-up (months)**	**Mortality**	**Analyses**
Yu, 2019 ^11^	22.9% ^1^	33.33%	NR	NR	Lung: 55.6% Liver: 22.2%	Range: 0.5-19	Mortality: 100% ^2^	NR
Zhang, 2019 ^8^	47.6%	NR	26.2%	NR	Distant: 29.2%	NR	Mortality: 85.3% Median DSS: 7.2 months	Worse DSS: ≥ 45 years, > 5 cm tumors Better DSS: surgery and radiotherapy ^3^
Leduc, 2017 ^19^	90%	30%	10%	30%	Distant: 50% Bone (20%), lung (20%), liver (10%), brain (10%)	4.4 (range: 1.2-61.2)	Mortality: 80% Median survival: 5.2 months	Chemoradiotherapy increased survival (p = 0.002) ^4^
Kupsky, 2016 ^12^	59%	47.1%	NR	29.4%	Distant: 76%	10 (range: 7-32) ^5^	Mortality: 64.7%	Surgical debulking group had a better survival benefit vs. conservative management (p = 0.25) TTE sensitivity: 75%
Look Hong, 2012 ^10^	94% ^6^	83.3%^7^	EBRT: 55.6% (palliative EBRT: 22.2%)	NR	Distant: 55.5% Lung (27.8%), liver (16.7%), bone (16.7%), spleen (11.1%), brain (5.6%)	12 (range: 1-77)	Mortality: 83.3% Median survival: 13 months Median survival in local PCA: 19.5 months Median survival in metastasis: 6 months	Surgery improved survival (p = 0.01)
Ge, 2011 ^18^	90% ^8^	100%	NR	NR	Distant: 80% Lung (50%), liver (20%), bone (10%), adrenal gland (10%), brain (10%), pleura (10%)	NR	Mortality: 90% Mean survival: 26.6 months Mean survival in regional extension: 51 months Mean survival in metastasis: 10.3 months	NR

Abbreviations: NR, not reported; DSS, disease-specific survival; TTE, transthoracic echocardiography; EBRT: external-beam radiotherapy; PCA: primary cardiac angiosarcoma. ^1^ They received radical resection due to small tumors. The rest of patients did not receive surgical treatment due to extensive metastases. ^2^ Two patients were lost to follow-up. No cases showed long-term survival. ^3^ The effect of surgical was marginal (HR: 1.427, 95% CI: 0.946-2.153) after adjusting for confounders. ^4^ However, patients in this group were younger. ^5^ Calculated using Stata v14. ^6^ Among them, 50% had R1 resection. ^7^ Single regimens: MAID (mesna, doxorubicin, ifosfamide, dacarbazine) or gemcitabine/docetaxel (n = 5), liposomal doxorubicin (n = 1), liposomal doxorubicin/paclitaxel (n = 1), doxorubicin/ifosfamide/mesna (n = 2), gemcitabine/docetaxel (n = 1), and doxorubicin/dacarbazine (n = 1). The rest received combinations of the regimens. ^8^ Surgical margins were positive in all patients.

 Patients with PCA had a very poor prognosis. Distant metastasis was common (5 studies: 29.2%-80%),^8,10,12,18,19^ and the most frequent locations were the lung (4 studies: 20%-55.6%),^10,11,18,19^ liver (4 studies: 10%-22.2%),^10,11,18,19^ and bone (3 studies: 10%-20%).^10,18,19^ Mortality ranged from 64.7% to 100%. Three studies reported a low survival in the entire group (5.2-26.6 months),^10,18,19^ and it was significantly lower in patients with metastases compared to local disease (2 studies, difference: 13.5% & 40.7%).^10,18^ (Table 2).

 One study suggested that transthoracic echocardiography was the best tool for diagnosis (sensitivity: 75%).^12^ Authors suggested that surgery (3 studies), and chemo/radiotherapy (2 studies) improved survival.^8,10,12,19^ One study reported that being ≥ 45 years, and having > 5 cm tumors were associated with lower survival. ^8^ (Table 1).

 The case series presented its clinical information in a reliable fashion per inclusion criteria (Table 3). In addition, the cohort study presented a high-quality score (8/9 points).^8^

**Table 3:** Quality assessment of case series

**Study**	**Clear inclusion criteria**	**Standard way of measurement condition**	**Valid method of identification**	**Consecutive inclusion **	**Complete inclusion**	**Demographics reporting**	**Clinical reporting**	**Follow-up/outcomes reporting**	**Presenting site demographics reporting**	**Appropriate analysis**
Yu, 2019^11^	Yes	Yes	Unclear	Yes	Unclear	No	Unclear	Yes	No	NA
Leduc, 2017^19^	Yes	Yes	Yes	Yes	Unclear	Unclear	Yes	Yes	Yes	Yes
Kupsky, 2016^12^	Yes	Yes	Yes	Yes	Yes	Unclear	Yes	Yes	Yes	Yes
Look Hong, 2012^10^	Yes	Yes	Yes	Yes	Unclear	Yes	Yes	Yes	Yes	NA
Ge, 2011^18^	No	Yes	Yes	Yes	No	Unclear	Yes	Yes	Yes	NA

Abbreviations: NA, not applicable.

## Discussion

###  Main findings

 Overall, we found that PCA are rare tumors with non-specific symptoms that often results in late diagnosis, which contributes in part to the related high mortality. They typically present in the right atrium and have an approximately equal to slight male predominance. The majority of patients present with metastasis at the time of diagnosis. There is no consensus on treatment, but options include surgery, chemotherapy, and radiation. Surgery with adjuvant chemotherapy may enhance the mean survival as well as decrease the percentage of distant relapses.

###  Age, sex, clinical manifestations

 In regards to gender distribution, the patients with PCA was approximately equal with a slight male predominance. Patients tended to be middle-aged with a mean/median age ranging from 39 to 48.9 years. The most typical manifestations of PCA were chest pain, dyspnea, and pericarditis/pericardial effusion. Given the relatively young age of presentation and the non-specific nature of the symptoms, the lack of early detection of these tumors has devastating consequences because at the time of presentation most patients have metastatic disease.

###  Imaging findings: CT scan, echocardiography

 On CT imaging, PCA often presents as heterogeneous centripetal enhancement. Some patients exhibit rapid inhomogeneous enhancement.^11^ Extension into the right ventricle, superior vena cava, and pericardium are also observed in some patients.^10,11^ Tumors are often described as non-mobile with broad-based attachment to the endocardium and smooth intra-cardiac borders when viewed with echocardiography. One study showed that up to 71% of patients have pericardial extension. By echocardiography, Kupsky DF et al found that 100% of patients exhibited hemodynamic obstruction, but 94% had preserved left ventricular ejection fraction (LVEF).^12^

###  Pathology: size, location, histological grade, immunohistochemistry

 Tumors were most commonly located in the right atrium and ranged in diameter from 5.8 to 7.2 cm. One cohort study showed 15.5% of patients had histological grade 3 tumors, 17.3% had grade IV tumors, and 26.2% exhibited regional disease.^8^ One case series showed that spindle cells and necrosis are common histological features^19^. Common immunohistochemistry markers for PCA include CD34 + , CD31 + , FLI-1 + , CD117 + , Ki-67 + , WT-1 + , and p53 + .^10,11,18^

###  Surgery, Chemotherapy, and radiotherapy

 Radical resection was a typical treatment choice and was commonly employed with adjuvant chemotherapy. In these studies, 33.3-100% of patients received adjuvant therapy. One study used neoadjuvant chemotherapy as a treatment option in 11.1% of patients.^10^ Radiotherapy was utilized in three studies in 11-26.2% of patients. Distant metastasis is a common occurrence with liver and lung being the most common locations. This can potentially limit treatment options to chemotherapy. Two studies’ analyses showed that surgery was associated with improving survival, but one study found a non-significant effect. Two studies concluded that radiotherapy/chemotherapy also improved survival. Overall, more work is needed to determine which patients will benefit most from which regimen. It appears that intervention is superior to observation alone; however, this must be balanced with the toxicities associated with treatment in individual patients.

###  Metastasis, mortality, and prognostic factors

 The most common metastasis location in this neoplasm is the lung, which shows multiple metastatic nodules. ^11^ Recurrence and metastasis from PCA are usually found by CT scan, magnetic resonance imaging, or PET/CT imaging. ^11^ Despite surgery achieving negative margins, patients with PCA usually have local recurrence, for this reason, radical resection has been studied, but similar survival rates were obtained. ^20-22^

 As explained previously, mortality of these patients is very high. However, there are promising results that report patients with local disease who underwent surgery and chemotherapy and achieved a mean survival of 51 months. ^18^ On the other hand, patients with distant metastasis usually have 5 to 12 months of survival. ^10,19^ While different treatment modalities have been studied, such as neo/adjuvant chemotherapy, surgical debulking, and radiotherapy, results are not consistent between studies; nevertheless, these modalities were found to be superior to conservative treatment. ^12,20,23^ Special consideration for toxicity from treatment should be assessed in those patients with metastatic disease.

###  Prognostic factors 

 In several types of cancer, age is an independent prognostic factor^24-26^ and also plays an important role in patients with PCA. In fact, patients older than 45 years have worse outcomes.^8^ In addition, Leduc et at.^19^ found that cytogenetic aberrations, such as 1q + , could have a prognostic relevance in PCA.^1^ According to Ge et al^18^, large tumors and regional invasion at surgery are associated with worse survival; this finding is supported by Zhang et al^8^, who stated that tumors greater than 5 cm have worse outcomes. Metastases at diagnosis affect the survival probability. Regarding treatment, surgical resection, even without clear margins, and adjuvant chemotherapy may improve survival.^18^ Although Zhang et al reported that radiotherapy increased the disease-specific survival, there are no further cohort studies that support this modality of treatment; however, some previous reports have shown promising results.^8,27,28^

 Currently, literature shows that histological grade, tumor necrosis, and lymph node metastasis are independent prognostic factors, whose explanation could be due to its hematogenous metastasis and advanced disease at presentation.^8,20,29^ These prognostic factors could be employed for a better approach, management, and multidisciplinary discussion to get better outcomes in spite of the aggressiveness of PCA.

 There are no previous systematic reviews that have synthesized evidence on PCA. Also, we systematically reviewed evidence through several databases. We extracted and described clinical information from several studies for a better understanding of PCA: presentation, imaging findings, and currently available treatments, in addition to the outcomes of those who underwent each modality. On the other hand, one limitation is that there was not enough data to perform an adequate meta-analysis since data mainly came from case series. Another limitation is that most studies were performed in a single institution, and the design is retrospective, so external validation would be reduced. Another important limitation is the high heterogeneity between the studies, which is reflected in different populations and number of patients.

## Conclusion

 PCA are very rare neoplasms and symptoms are mainly nonspecific, often resulting in late diagnosis. The most frequent location is the right atrium. Most PCAs have metastases at diagnosis, as well as very high mortality; hence, early detection is very important for a better assessment of the different available therapies. Currently, multiple studies have shown that surgery and adjuvant chemotherapy limit the growth and spread of this neoplasm; indeed, both may enhance the mean survival. New oncological markers and diagnostic modalities are needed for earlier diagnosis and better follow-up of these patients. We recommend performing multi-center prospective cohorts to support better information for consensus, algorithms, and guidelines for this type of sarcoma.

## Acknowledgments

 None.

## Competing Interests

 Rosalie M. Sterner is an inventor on patents and royalties in the field of CAR-T cell therapy licensed to Humanigen through Mayo Clinic.

## Ethical Approval

 This systematic review did not collect new data. All data is publicly available in the included primary studies, therefore this systematic review did not require ethical approval.

## Funding

 Self-funded.

## Supplementary Files

Supplementary material consists of search strategies.
