# Supplementary material for: Clinical characteristics, management, and outcomes of patients with primary cardiac angiosarcoma: A systematic review
Source: J Cardiovasc Thorac Res. 2023 Mar 16;15(1):1–8. doi: 10.34172/jcvtr.2023.30531 (PMC10278191; doi:10.34172/jcvtr.2023.30531)
Supplement: Supplementary file 1 — Supplementary material consists of search strategies. [file jcvtr-15-1-s001.pdf]

J Cardiovasc Thorac Res, 2023, 15(1), 1-8
doi: 10.34172/jcvtr.2023.30531
http://jcvtr.tbzmed.ac.ir

Clinical characteristics, management, and outcomes of patients with primary cardiac angiosarcoma: A systematic review

Diego Chambergo-Michilot[1,2], Gabriel De la Cruz-Ku[1,3,4*], Rosalie M. Sterner[3], Ana Brañez-Condorena[5,6], Pedro Guerra-Canchari[5,7], John Stulak[3]

[1]Universidad Científica del Sur, Lima, Perú
[2]Department of Cardiology Research, Torres de Salud National Research Center, Lima, Perú
[3]Department of Surgery, Mayo Clinic, Rochester, MN, USA
[4]Department of Surgery of the University of Massachusetts Medical School, Worcester, MA, USA
[5]Universidad Nacional Mayor de San Marcos, Facultad de Medicina, Lima, Perú
[6]Asociación de Investigación Estudiantil en Ciencias de la Salud, Lima, Perú
[7]Sociedad Científica de San Fernando, Lima, Perú

Supplementary material 1. Search strategies.

PubMed

#1: heart[MESH] OR hearts[mesh] OR myocardium[mesh] OR Cardiac Muscle[mesh] OR Muscle, Heart[mesh] OR Heart Muscle[mesh] OR Heart Muscles[mesh] OR Muscles, Heart[mesh] OR Myocardia[mesh] OR Muscle, Cardiac[mesh] OR Cardiac Muscles[mesh] OR Muscles, Cardiac[mesh] OR endocardium[mesh] OR endocardiums[mesh] OR heart[tiab] OR hearts[tiab] OR myocardium[tiab] OR “Cardiac Muscle”[tiab] OR “Heart Muscle”[tiab] OR “Heart Muscles”[tiab] OR Myocardia[tiab] OR “Cardiac Muscles”[tiab] OR cardiac[tiab] OR endocardium[TIAB] OR endocardiums[TIAB] OR “heart muscle structure”[tiab] OR “heart muscle volume”[tiab] OR “myocardial muscle”[tiab] OR “myocardial structure”[tiab] OR “myocardium extract”[tiab] OR “myocardium structure”[tiab] OR endocard[tiab] OR endocardial[tiab] OR cor[tiab] OR “cardiac effect”[tiab] OR “myocardial tissue”[tiab] OR heart[OT] OR hearts[OT] OR myocardium[OT] OR cardiac muscle[OT] OR Muscle, Heart[OT] OR Heart Muscle[OT] OR Heart Muscles[OT] OR Muscles, Heart[OT] OR Myocardia[OT] OR Muscle, Cardiac[OT] OR Cardiac Muscles[OT] OR Muscles, Cardiac[OT] OR endocardium[OT] OR endocardiums[OT] OR muscle, heart[OT] OR heart muscle structure[OT] OR heart muscle volume[OT] OR myocardial muscle[OT] OR myocardial structure[OT] OR myocardium extract[OT] OR myocardium structure[OT] OR endocard[OT] OR endocardial[OT] OR cor[OT] OR cardiac effect[OT] OR myocardial tissue[OT]

#2: hemangiosarcoma[MESH] OR hemangiosarcomas[MESH] OR angiosarcoma[MESH] OR angiosarcomas[MESH] OR haemangioendothelioma, malignant[MESH] OR hemangioendothelioma, malignant[MESH] OR angiosarcoma[tiab] OR angiosarcomas[tiab] OR angioendotheliosarcoma[tiab] OR haemangiosarcoma[tiab] OR “hemangio endothelial sarcoma”[TIAB] OR “hemangio endotheliosarcoma”[TIAB] OR “hemangio sarcoma”[TIAB] OR “hemangioendothelial sarcoma”[TIAB] OR hemangioendotheliosarcoma[TIAB] OR hemangiosarcoma[TIAB] OR hemangiosarcomas[TIAB] OR “malignant haemangioendothelioma”[TIAB] OR “malignant hemangioendothelioma”[TIAB] OR angiosarcoma[OT] OR angiosarcomas[OT] OR angioendotheliosarcoma[OT] OR haemangiosarcoma[OT] OR hemangio endothelial sarcoma[OT] OR hemangio endotheliosarcoma[OT] OR hemangio sarcoma[OT] OR hemangioendothelial sarcoma[OT] OR hemangioendotheliosarcoma[OT] OR hemangiosarcoma[OT] OR hemangiosarcomas[OT] OR

malignant haemangioendothelioma[OT] OR malignant hemangioendothelioma[OT] OR haemangioendothelioma, malignant[OT] OR hemangioendothelioma, malignant[OT]

#1 AND #2

SCOPUS

TITLE-ABS-KEY((heart OR hearts OR myocardium OR “Cardiac Muscle” OR “Heart Muscle” OR “Heart Muscles” OR Myocardia OR “Cardiac Muscles” OR cardiac OR endocardium OR endocardiums OR “heart muscle structure” OR “heart muscle volume” OR “myocardial muscle” OR “myocardial structure” OR “myocardium extract” OR “myocardium structure” OR endocard OR endocardial OR cor OR “cardiac effect” OR “myocardial tissue”) AND (angiosarcoma OR angiosarcomas OR angioendotheliosarcoma OR haemangiosarcoma OR “hemangio endothelial sarcoma” OR “hemangio endotheliosarcoma” OR “hemangio sarcoma” OR “hemangioendothelial sarcoma” OR hemangioendotheliosarcoma OR hemangiosarcoma OR hemangiosarcomas OR “malignant haemangioendothelioma” OR “malignant hemangioendothelioma”))

WOS

#1 KP=(“Muscles, Heart” OR “Muscle, Cardiac” OR “Muscles, Cardiac” OR heart OR hearts OR myocardium OR “Cardiac Muscle” OR “Heart Muscle” OR “Heart Muscles” OR Myocardia OR “Cardiac Muscles” OR cardiac OR endocardium OR endocardiums OR “heart muscle structure” OR “heart muscle volume” OR “myocardial muscle” OR “myocardial structure” OR “myocardium extract” OR “myocardium structure” OR endocard OR endocardial OR cor OR “cardiac effect” OR “myocardial tissue”) OR AK=(“Muscles, Heart” OR “Muscle, Cardiac” OR “Muscles, Cardiac” OR heart OR hearts OR myocardium OR “Cardiac Muscle” OR “Heart Muscle” OR “Heart Muscles” OR Myocardia OR “Cardiac Muscles” OR cardiac OR endocardium OR endocardiums OR “heart muscle structure” OR “heart muscle volume” OR “myocardial muscle” OR “myocardial structure” OR “myocardium extract” OR “myocardium structure” OR endocard OR endocardial OR cor OR “cardiac effect” OR “myocardial tissue”) OR TI=(“Muscles, Heart” OR “Muscle, Cardiac” OR “Muscles, Cardiac” OR heart OR hearts OR myocardium OR “Cardiac Muscle” OR “Heart Muscle” OR “Heart Muscles” OR Myocardia OR “Cardiac Muscles” OR cardiac OR endocardium OR endocardiums OR “heart muscle structure” OR “heart muscle volume” OR “myocardial muscle” OR “myocardial structure” OR “myocardium extract” OR “myocardium structure” OR endocard OR endocardial OR cor OR “cardiac effect” OR “myocardial tissue”)

#2 KP=(angiosarcoma OR angiosarcomas OR angioendotheliosarcoma OR haemangiosarcoma OR “hemangio endothelial sarcoma” OR “hemangio endotheliosarcoma” OR “hemangio sarcoma” OR “hemangioendothelial sarcoma” OR hemangioendotheliosarcoma OR hemangiosarcoma OR hemangiosarcomas OR “malignant haemangioendothelioma” OR “malignant hemangioendothelioma” OR “haemangioendothelioma, malignant” OR “hemangioendothelioma, malignant”) OR AK=(angiosarcoma OR angiosarcomas OR angioendotheliosarcoma OR haemangiosarcoma OR “hemangio endothelial sarcoma” OR “hemangio endotheliosarcoma” OR “hemangio sarcoma” OR “hemangioendothelial sarcoma” OR hemangioendotheliosarcoma OR hemangiosarcoma OR hemangiosarcomas OR “malignant haemangioendothelioma” OR “malignant hemangioendothelioma” OR “haemangioendothelioma, malignant” OR “hemangioendothelioma, malignant”) OR TI=(angiosarcoma OR angiosarcomas OR angioendotheliosarcoma OR haemangiosarcoma OR “hemangio endothelial sarcoma” OR “hemangio endotheliosarcoma” OR “hemangio sarcoma” OR “hemangioendothelial sarcoma” OR hemangioendotheliosarcoma OR hemangiosarcoma OR hemangiosarcomas OR “malignant haemangioendothelioma” OR “malignant hemangioendothelioma” OR “haemangioendothelioma, malignant” OR “hemangioendothelioma, malignant”)

#1 AND #2

EMBASE

#1 'endocardium':de OR 'endocard':tn,ti,ab OR 'endocardial':tn,ti,ab OR 'endocardium':tn,ti,ab

#2 'cardiac muscle':de OR 'cardiac muscle':tn,ti,ab OR 'heart muscle':tn,ti,ab OR 'heart muscle structure':tn,ti,ab OR 'heart muscle volume':tn,ti,ab OR 'muscle, heart':tn,ti,ab OR 'myocardial muscle':tn,ti,ab OR 'myocardial structure':tn,ti,ab OR 'myocardium':tn,ti,ab OR 'myocardium extract':tn,ti,ab OR 'myocardium structure':tn,ti,ab

#3 'heart':de OR 'cardiac effect':tn,ti,ab OR 'cor':tn,ti,ab OR 'heart':tn,ti,ab OR 'myocardial tissue':tn,ti,ab

#4 'angiosarcoma':de OR 'angioendotheliosarcoma':tn,ti,ab OR 'angiosarcoma':tn,ti,ab OR 'haemangioendothelioma, malignant':tn,ti,ab OR 'haemangiosarcoma':tn,ti,ab OR 'hemangio endothelial sarcoma':tn,ti,ab OR 'hemangio endotheliosarcoma':tn,ti,ab OR 'hemangio sarcoma':tn,ti,ab OR 'hemangioendothelial sarcoma':tn,ti,ab OR 'hemangioendothelioma, malignant':tn,ti,ab OR 'hemangioendotheliosarcoma':tn,ti,ab OR 'hemangiosarcoma':tn,ti,ab OR 'malignant haemangioendothelioma':tn,ti,ab OR 'malignant hemangioendothelioma':tn,ti,ab

(#1 OR #2 OR #3) AND #4
